# Genomic profiling of *Morganella morganii*: insights into antibiotic resistance genes, pathogenicity islands, and toxin-antitoxin systems

**DOI:** 10.1128/spectrum.02207-25

**Published:** 2026-05-12

**Authors:** Tosin Yetunde Senbadejo, Christina R. Bourne, Abiola Isawumi

**Affiliations:** 1 West African Centre for Cell Biology of Infectious Pathogens (WACCBIP), Department of Biochemistry, University of Ghana, Accra, Ghana; 2 Department of Chemistry and Biochemistry, University of Oklahoma, Norman, Oklahoma, USA; 3 Antimicrobial Resistance Research Group, West African Centre for Cell Biology of Infectious Pathogens, College of Basic and Applied Science, University of Ghana 58835, Legon Accra, Ghana; University of Pretoria 56410, Pretoria, Gauteng, South Africa

**Keywords:** toxin-antitoxins, defense systems, *Morganella morganii*, virulence, antimicrobial resistance, pathogenicity islands

## Abstract

*Morganella morganii* is an opportunistic pathogen affecting immunocompromised and hospitalized intensive care patients. It colonizes diverse environments and exhibits multidrug resistance. Its genome encodes factors that impart pathogenicity and resistance to multiple antibiotics. This study provided insights into the resistome, mobilome, and toxin-antitoxin systems of *M. morganii* strains from human clinical samples, food, animals, and wastewater. The pangenome predicted 2,852 core genes shared by >99% of the strains, with >30% of coding sequences annotated as hypothetical proteins co-localizing with virulence factors on mobile genetic elements (MGEs). *M. morganii* carried 1–3 plasmids and up to 71 diverse MGEs, independent of strain source. The strains shared unique 13–33 GI clusters (10–167 kb) in chromosomes and plasmids harboring transposable elements, phage-associated proteins, toxin-antitoxins (TAs), and additional virulence determinants. Diverse antimicrobial resistance genes, including *aac(3)-lld, bla_KPC_,* and *bla_NDM_,* were identified. A total of 24 distinct TA families were identified, predominantly belonging to the type II group, and three were identified as types IV and VII. The *hha-tomB, higBA, mazEF, vapBC,* and *doc-phd* TAs were the most prevalent, typically occurring as a single copy (*hha-tomB*) or multiple copies (*higBA*) in all strains. Although the presence or absence of plasmid and other mobile genetic elements influences the number of resistance genes, it has less of an effect on toxin-antitoxin system distribution. These findings emphasize the significance of MGEs in the evolution and propagation of antimicrobial resistance genes and virulence factors within *M. morganii*, highlighting their contribution to bacterial adaptation and survival in hostile environments.

**IMPORTANCE:**

*Morganella morganii*, an emerging opportunistic pathogen, has garnered increasing attention in recent years due to its role in nosocomial infections and rising antimicrobial resistance (AMR). This study provides a comprehensive genomic analysis of *M. morganii* strains from diverse sources, revealing how mobile genetic elements (MGEs) drive the spread of AMR genes and virulence factors. By mapping the resistome, mobilome, and toxin-antitoxin (TA) systems across the pangenome, we uncover the conserved and variable genomic features that enable *M. morganii* to thrive in clinical and environmental niches. Our findings offer novel insights into the potential genomic determinants of persistence, adaptation, and multidrug resistance in an understudied and emerging opportunistic pathogen, informing surveillance strategies and the development of targeted interventions. This study shows that *M. morganii* encodes chromosomal and mobile genetic element-mediated virulence factors. Resistance and virulence determinants in *M. morganii* are comparable to those of other known pathogenic bacteria; hence, they should be given significant research attention.

## INTRODUCTION

*Morganella morganii* is an emerging opportunistic pathogen associated with community and hospital-acquired infections (1). *M. morganii* causes sepsis (2), abscess (3), bloodstream (4), and urinary tract infections (5), particularly in immune-compromised individuals with underlying comorbidities (6). As a histamine-producing bacterium, it contaminates food and causes food-borne histamine poisoning (7). *M. morganii* can also be zoonotic, causing infections in a diverse range of animals (8). *M. morganii* causes severe infections with high mortality rates, and this is compounded by the lack of effective antibiotics (9). *M. morganii* possesses *ampC* and *ampR* genes that confer intrinsic resistance to ampicillin, amoxicillin, and first- and second-line cephalosporins (10). Resistance genes (RGs) are also acquired via plasmids and transposons (or integrons), further contributing to their increasing resistance levels (11). This poses a significant concern in clinical treatment, as reflected by the WHO 2024 designation as a priority pathogen, necessitating a need to better explore the genome of *M. morganii* strains (5).

*M. morganii* strains have virulence factors that enable their survival in hostile environments, such as in wounds, respiratory, and urinary tracts. Urinary tract infections caused by *M. morganii* are common in patients with indwelling catheters and/or those in long-term care facilities (nursing homes) (12). Severe infections associated with long-term catheterization result from polymicrobial interactions and the formation of biofilms (13). Pathogenic bacteria, such as *Escherichia coli*, *Proteus mirabilis*, *Klebsiella pneumoniae*, and *Pseudomonas aeruginosa*, have also been implicated in polymicrobial infections with high levels of intrinsic resistance and expression of virulence factors (14). *Morganella* possesses a diverse array of virulence factors that contribute to their ability to colonize (8), evade host immune systems, and increase their disease spectrum (1). For instance, these virulence factors play essential roles in facilitating bacterial colonization, invasion, and damage to host tissues in urinary tract infections (15).

Virulence and resistance determinants are harbored and spread by mobile genetic elements (MGEs) (16), including transposons and insertion sequences (which mediate gene movement within a genome), conjugative elements, such as plasmids and integrative conjugative elements (which transfer genes between cells via conjugation), and bacteriophages (which disseminate genes between different cells via transduction) (17). These gene sets are usually harbored in a genomic region known as genomic islands (GIs). They are classified based on their advantageous functions, such as resistance, virulence, metabolism, or symbiosis, in bacteria (18). Pathogenicity islands (PAIs), a specific type of genomic island that facilitates the horizontal gene transfer (HGT) of virulence factors, are usually harbored by pathogenic bacteria (19). Such virulence-associated genes encompassing fimbriae, ureases, hemolysin, insecticidal toxins, type III secretion systems, proteases, flagella, and siderophores have been identified within the annotated genomes of clinical *M. morganii* strains (9, 20). GIs play an essential role in the adaptation and evolution of bacterial genomes.

As a part of their genome profiles, bacteria have toxin-antitoxin (TA) systems, which are self-regulating genetic elements ubiquitously present in bacterial chromosomes and plasmids, often in multiple copies (21). They function as effectors of stress response systems and consist of two main components: a toxin that inhibits essential cellular processes, such as DNA replication, translation, membrane integrity, and cell wall biosynthesis, and a cognate antitoxin that neutralizes the toxin’s activity (22, 23). These systems can potentially contribute to their virulence and antibiotic tolerance in hostile environments. TA systems have been extensively studied in *E. coli* (24), *P. aeruginosa* (25), *P. mirabilis* (26), and *K. pneumoniae* (27). Some studies have reported that TA systems may be involved in the formation of persister cells (28), biofilms (29), and defense against phages (30). Also, TA systems have roles in the maintenance of plasmids that can confer antibiotic resistance on bacteria (31, 32). However, the role of TA systems in persister formation remains controversial, as other studies have questioned or refuted a direct causal relationship, suggesting that their contribution may be context-dependent or indirect (33). Relatedly, recent studies have shown that *M. morganii* harbor diverse phages that can infect different hosts (34–36).

To study the factors that could potentially be contributing to the survival of *M. morganii* in different hosts and environments, *in silico* genomic profiling was conducted. Complete genome sequences of 35 *M. morganii* (File S1) from the NCBI database were used to profile TAs, RGs, and other virulence factors located within the GIs. Correlating these virulence factors with existing knowledge may provide insights into new therapeutic and diagnostic approaches to alleviate infections by *M. morganii*.

## RESULTS

### *M. morganii* strains are from diverse sources

There are 1,078 whole-genome sequences of *M. morganii* deposited in the NCBI database as of March 2025. These include isolates from humans, animals, food, and environmental sources. Most of the strains (60%) are from China, while the remaining 40% cut across strains from the United Kingdom, the United States, Taiwan, Switzerland, Canada, the Czech Republic, India, and Korea. Of the 1,078, only 73 of these genomes are complete and have been curated with designated RefSeq accession numbers. To obtain a representative subset of these genomes, strains with redundant metadata, such as those derived from the same BioProject, geographic location, or isolation source, and MGEs were excluded, as well as strains lacking source or collection information. A total of 35 complete RefSeq genomes were ultimately selected for this study based on year of collection, sources, and geographic origin (File S1). The selected genomes originated from diverse geographic regions, with isolates from Asia, Europe, and North America. China accounted for the majority of the genomes (*n* = 21), followed by the United States (*n* = 3), Switzerland (*n* = 2), Taiwan (*n* = 2), and single isolates from the United Kingdom, France, South Korea, the Czech Republic, Canada, and India. One human gut isolate had only “EBI” listed as its location in NCBI, so its geographic origin could not be determined. Although the data set is enriched for isolates from China, this reflects the current availability of complete *M. morganii* genomes in public databases rather than a deliberate sampling bias, and the inclusion of isolates from multiple continents provides a broad overview of the species’ genomic features.

The genomes ranged in size from 3.6 to 4.4 Mb with an average GC content of approximately 51%. Approximately 51% of the selected genomes were isolated from clinical human samples, including urine, blood, adenocarcinoma tissue, stool, rectal swab, and sputum. Animal-derived isolates accounted for 25% of the genomes and originated from a range of hosts, including mink, pig, poultry, cattle, snake, frog, and *Manis javanica*. The remaining 25% comprised isolates from food sources, mainly fish and cheese, as well as wastewater samples (File S1).

### *M. morganii* strains have MGEs that harbor TA systems, resistance, and virulence factors

**Fig 1:**
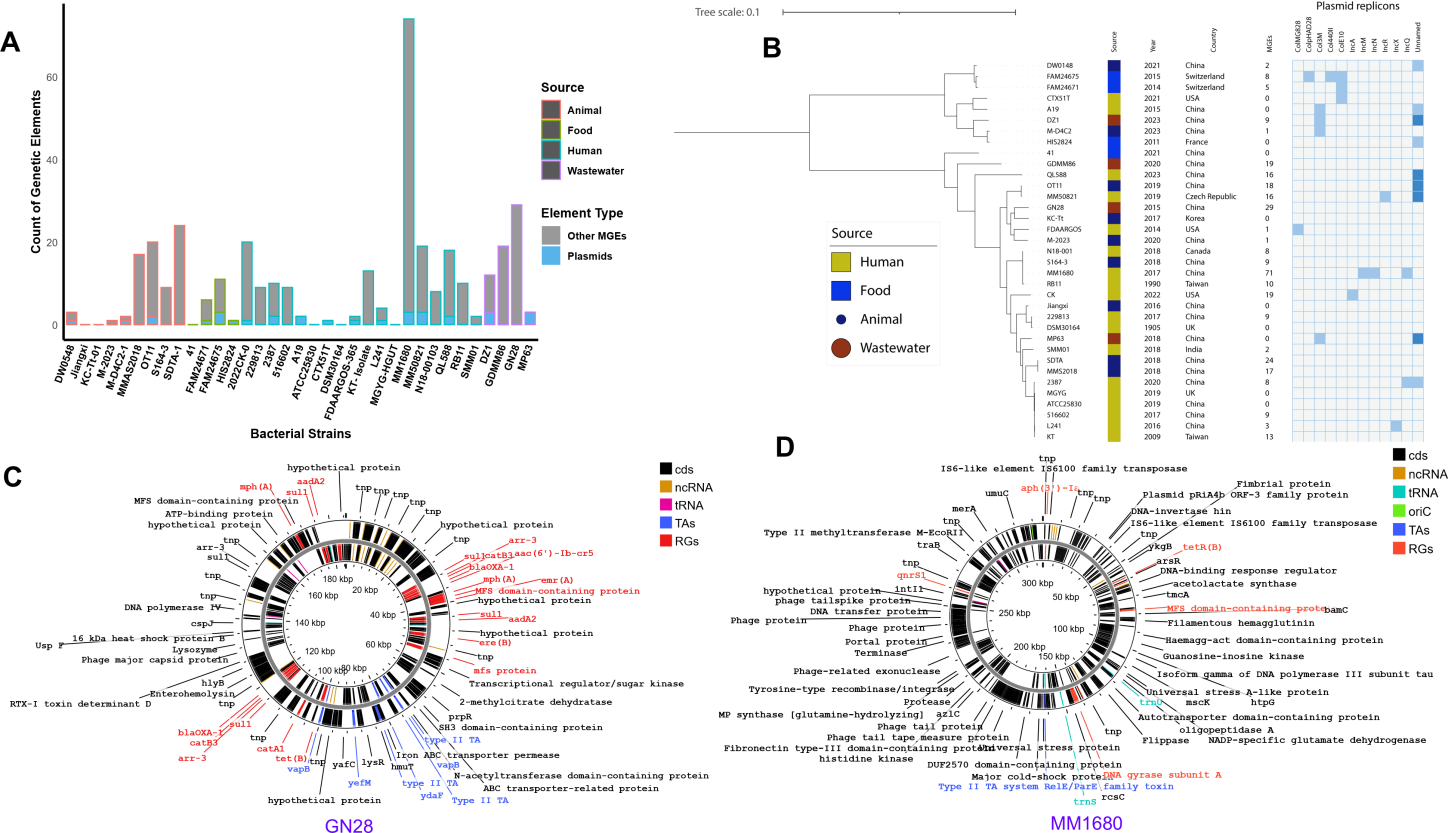
Distribution and characterization of MGEs in *M. morganii* strains. (**A**) Frequency of genetic elements per strain, based on the source of isolation, shows limited correlation between strains. (**B**) A midpoint-rooted maximum likelihood phylogenetic tree constructed from the sequence alignment of 2,852 core genes in the 35 *M. morganii* strains demonstrates genetic diversity relatively independent of isolation source, year, or location. Each strain was also annotated with its corresponding isolation source, year, or location, and number of MGEs. The heatmap shows the assigned plasmid replicon type, color-coded as different shades of blue, indicating the presence of plasmid replicon type, color-coded from 1 (light blue) to 3 (deep blue), and the colorless squares represent the absence of plasmids in the strains. The tree scale indicates the number of nucleotide substitutions per site. (**C and D**) Annotation of MGE sequences of GN28 and MM1680, respectively.

The MGEs comprise plasmids and transposable elements, including insertion sequence elements, units, and composite transposons in the genome of the strains. The *M. morganii* genome encoded a high number of MGEs, ranging from 1 to 71 per genome, with up to three plasmids identified per genome (Fig. 1A). Approximately 46% (16/35) of the strains carried plasmids, and 71% have transposable elements, while 17% have neither plasmids nor transposable elements. Of the 18 human clinical-originated strains, 9 do not possess plasmids, while 7 encode both plasmids and transposable elements. The genome with the highest number of plasmids (*n* = 3) and transposable elements (*n* = 71) was identified in strain MM1680 isolated from human (sputum) in China. Another genome, denoted as strain GN28, is an isolate from wastewater in China harboring 29 transposable elements but no plasmids. The plasmid replicon types identified in the strains include Incompatibility groups (Inc) Family (IncA, IncM, IncN, IncR, IncQ, IncX), and the Colicinogenic (Col) family (ColMG828, Col440II, ColE10, ColpHAD28), although some remain unnamed. Only seven strains of human, wastewater, and animal origin harbored multiple plasmids (Fig. 1B). Some of these plasmids, irrespective of isolation source, also carried similar transposable elements as those found in chromosomes. The plasmids in the strains also harbor prophages, phage-associated proteins (PAPs), transposable elements, secretion systems, TA systems, restriction enzymes, and stress-associated proteins (File S2). The transposable elements and plasmids also encode shared and core RGs, suggesting transfer of genes within bacteria. The most occurring transposable elements that appeared in multiple numbers and in more than 50% of the strains are insertion sequence elements IS26 and ISVsa5 and their composite transposon elements (cn_IS26, cn_ISVsa5) (File S3). To have a holistic view of the virulence factors harbored by the MGEs, strains with the highest number of MGEs (GN28, an environmental [wastewater-derived] isolate, and MM1680, a clinical human-derived isolate) were selected for further MGE annotation. The MGE sequences from each strain were concatenated and annotated using BAKTA v1.8.2 on Proksee v1.1.0 (37) and TADB to screen for antimicrobial resistance (AMR), virulence-associated genes, and TA systems. In GN28, MGEs encoded multiple AMR genes, including *catB3*, *blaOXA-1*, *mph(A*), *aadA2*, and *ere(B*), as well as stress-response proteins (heat shock protein, universal stress protein), virulence-associated factors (RTX-I toxin determinant, enterohemolysin, *hlyB*, *yafC*), prophage-associated genes, transposases, hypothetical proteins (HPs), and multiple TA systems (Fig. 1C).

Similarly, MGEs in MM1680 carried resistance genes [*aph(3*′*)-Ia*, *qnrS1*, *gyrA*, and MFS efflux pumps], stress-response proteins (cold shock protein and universal stress protein), virulence-associated genes (filamentous hemagglutinin, type IV secretion system components, *umuC*, histidine kinase *rcsC/D*), phage-related proteins, kinases, HPs, and type II TA system (Fig. 1D).

### Pangenome profile indicates high genetic diversity among the *M. morganii* strains

The collective set of genes (pangenome), present in the genome of the strains used in this study, was characterized using Panaroo v.1.5 (38). Pangenome analysis of the selected *M. morganii* strains identified a total of 9,479 genes, with only 2,852 (30%) core genes shared by ≥99% of strains considered in this study, and 89 (∼1%) soft core genes present in 95%–99% of strains. The core and soft-core genes likely encode essential cellular functions conserved across the species. The accessory genome included 1,527 (16%) shell genes, found in 15%–95% of strains, and 5,011 (53%) cloud genes, which are the strain-specific genes present in fewer than 15% of strains (File S4). The high number of accessory and cloud genes suggests substantial genomic plasticity, and they may include strain-specific MGEs, prophages, virulence factors, and RGs that can contribute to environmental adaptation and host-specific interactions. The pangenome is indicative of frequent or active HGT and genetic diversification within the *M. morganii* species. In the *M. morganii* strains, the number of genes per genome ranges from 3,584 to 4,267. The smallest, KC-Tt-01, was isolated from fish in Korea, while the isolate DWO548 from poultry in China has the highest number of genes.

### The pangenome of *M. morganii* has a high number of HPs

**Fig 2:**
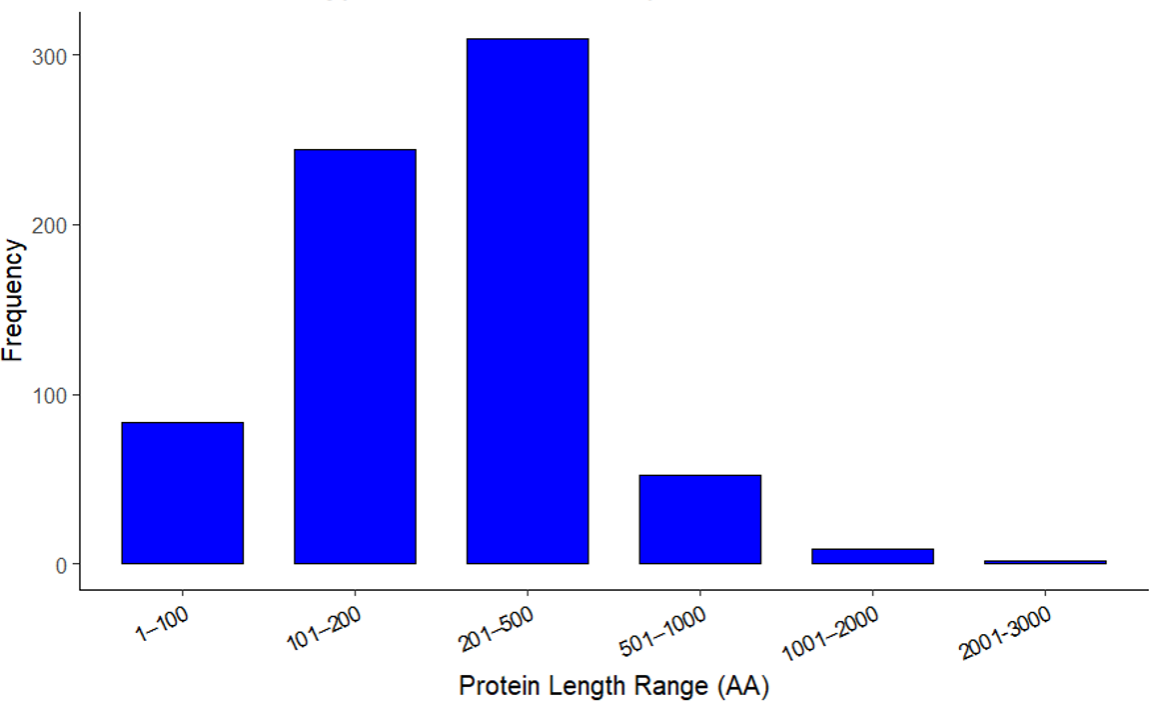
Distribution of HP length in core genes of the 35 analyzed *M. morganii* strains (for functional annotations, see File S5).

In the pangenome core-gene category, approximately 702 genes (~24%) were predicted as HPs. Additionally, 8%–11% of annotated genes in each genome were classified as HPs. For example, the reference *M. morganii* ATCC25830 strain contains 312 HPs out of 3,738 coding sequences. Overall, these HPs vary in amino acid (AA) lengths from a minimum of 30 to a maximum of 2,508 AA, with 34.7% having 100–200 AA and approximately 12% being small proteins (≤100 AA) (Fig. 2).

To assess their potential biological relevance, HP sequences from the core genome were extracted and re-annotated using Bakta, which employs advanced gene prediction and functional annotation algorithms, including Hidden Markov Models and machine learning, to assign functions based on conserved domains or sequence features. This analysis reassigned putative functions for more than 500 previously classified HPs based on detectable homology to characterized protein families. These HPs showed similarity to known proteins involved in virulence-associated processes, including fimbrial biogenesis, secretion systems, TA systems, adhesins, hemolysins, two-component regulatory systems, efflux pumps, and AMR determinants (File S5). Thus, the majority of annotated HPs do not represent novel functional classes, but rather divergent or previously uncharacterized homologs of established virulence- and adaptation-associated proteins. In contrast, a subset of HPs remained unannotated following domain-based and homology-driven analysis, suggesting that these proteins may represent either highly diverged homologs or potentially novel functions unique to *M. morganii*. The conservation of many HPs within the core genome further implies that they may play essential roles in physiology, stress adaptation, or host interaction, warranting future experimental characterization.

### *M. morganii* genomes harbor multiple genomic islands that encode resistance and virulence factors

**Fig 3:**
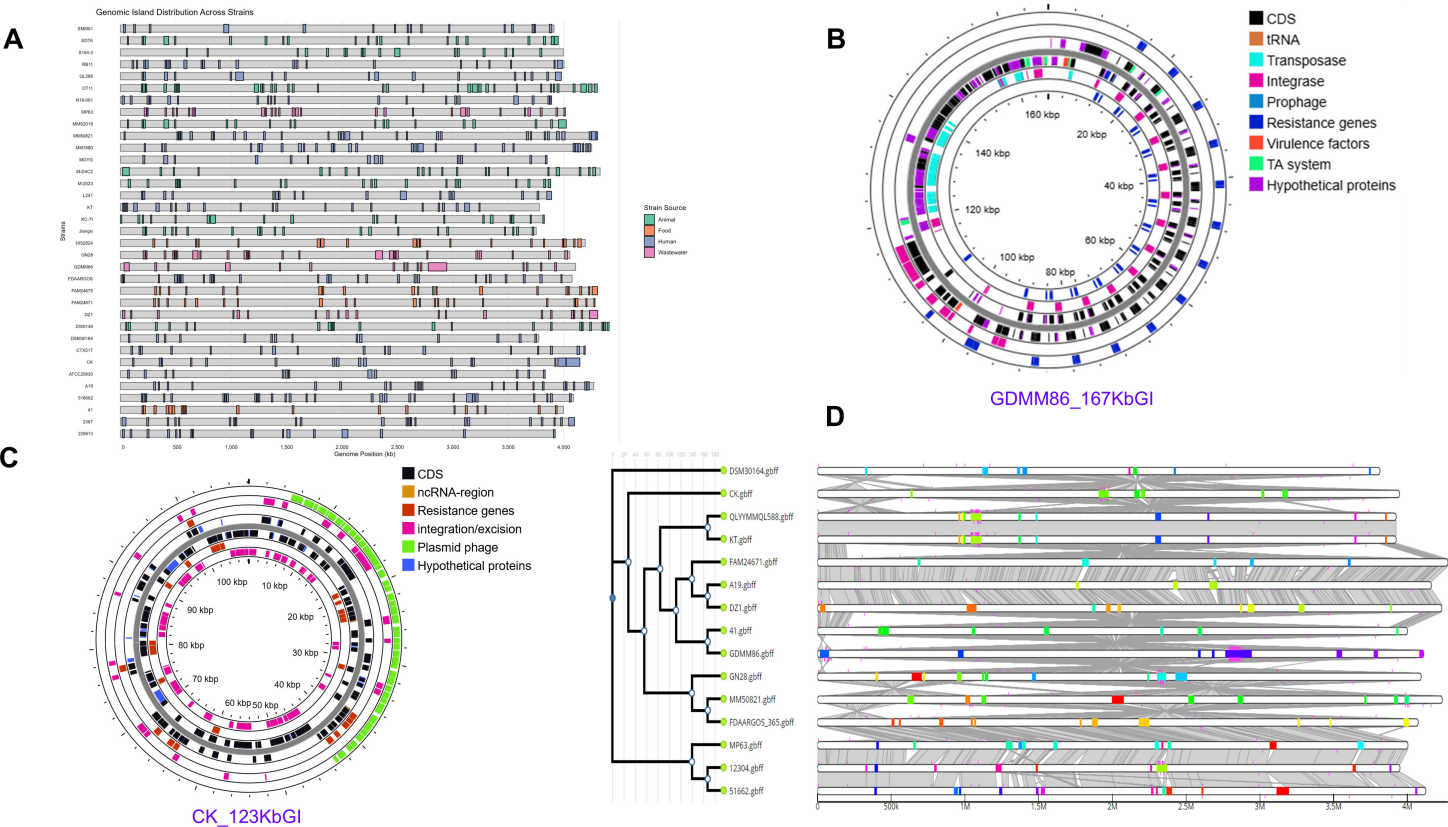
Genomic islands in *M. morganii* strains. (**A**) Number and size distribution of GI clusters across strains, color-coded based on the different sources. GIs ranged from 13 to 33 clusters per genome (chromosome and plasmids), with sizes from ~10 to ~167 kb. (**B**) Annotation of the largest GI cluster (~167 kb) from a wastewater isolate (GDMM86) using Bakta, Comprehensive Antibiotic Resistance Database (CARD) resistance gene identifier, Phigaro, TADB, and mobileOG-db. From the outermost to the innermost circle, the tracks represent coding sequences (CDSs; black), transfer RNA (tRNA; orange), transposase; bright light blue, integrase (pink), prophage (blue), antimicrobial resistance genes (RGs; dark blue), virulence factors (orange), TA systems (green), and hypothetical proteins (HPs; purple). The innermost scale indicates genomic coordinates in kilobase pairs (kbp). Resistance genes include multiple copies of *sul1, qacE, arr-3, catB, blaNDM-1, ble, mphA, aph,* and *mecA,* conferring resistance to seven antibiotic classes. (**C**) Circular map of a ~126 kb plasmid-encoded genomic island (GI) identified in *Morganella morganii* strain CK (human isolate). The map was generated using Proksee, and gene annotation was performed using Bakta, CARD RGI, Phigaro, and mobileOG-db. The concentric circles represent genomic regions, with multiple feature types distinguished by color: coding sequences (CDSs, black), non-coding RNA regions (ncRNA, orange), antimicrobial resistance genes (RGs, red), mobile genetic elements, including integrases, transposases, and excision genes (pink), plasmid- and phage-related genes (green), and hypothetical proteins (HPs, blue). The innermost scale indicates genomic coordinates in kilobase pairs (kbp). This GI harbors multiple antimicrobial resistance genes [blaCTX, blaTEM, blaOXA, aph(6′), aac(3′), aac(6′), tetA, sul2, and catB3], mobile genetic elements, and phage-associated genes, but lacks identifiable toxin–antitoxin systems. (**D**) Comparative GI cluster analysis. The homologous regions are denoted by gray shading, and GIs are denoted as colored clusters (blue, red, green, lemon, orange, purple) placed on a linear representation of the genome. Shared GI clusters across genomes that share sequence similarity have similar colors. The genomes are also overlaid with AMR determinants (predicted by the CARD webserver tool); the pink lines beneath the GI clusters indicate the positions in the genome where RGs are present.

GIs are large DNA segments acquired through HGT that integrate into bacterial genomes (39). They often carry genes that confer selective advantages, such as antibiotic resistance, novel metabolic capabilities, or environmental adaptation. In *M. morganii* strains, GI clusters are widely distributed throughout the genome, ranging from 13 to 33 clusters per strain, with sizes varying from approximately 10 to 167 kb (Fig. 3A). Human-derived isolates harbor between 13 and 29 GIs, animal isolates possess 17 to 33 GIs, food-associated isolates contain 19 to 22 GIs, and environmental isolates encode between 16 and 28 GI clusters. Only two strains exhibited GIs exceeding 100 kb in size. The largest GI cluster was identified in strain GDMM86, a wastewater isolate from China, followed by a 123 kb GI detected in strain CK, a human isolate from the United States.

The largest GI cluster (~167 kb) contained over 200 genes, including virulence factors, transposable elements, HPs, and resistance determinants (Fig. 3B). Multiple copies of *sul1*, *qacE*, *arr-3*, *catB*, *blaNDM-1*, *ble*, *mphA*, *aph*, and *mecA* were identified, conferring predicted resistance to seven antibiotic classes, including carbapenems. This region also encoded multidrug efflux pumps (*tap*), prophages, and TA systems (*prlf-yhaV*, *hip*, *vapB*, and GNAT-family acetyltransferase homologs). Transposable elements and HPs flanked resistance genes, indicating a high potential for mobility and horizontal gene transfer. Although the functions of HPs remain unclear, they may influence gene regulation, stability, or dissemination.

While GIs are predominantly chromosomal, a 123 kb island-like region was found on a 198 kb plasmid in strain CK, encoding RGs, transposable elements, siderophores, secretion systems, phage genes, and HPs but lacking TA systems (Fig. 3C; note, this plasmid sequence was appended to the genome sequence shown in Fig. 3A). Detected resistance genes [*blaCTX*, *blaTEM*, *blaOXA*, *aph(6*′), *aac(3*′), *aac(6*′), *tetA*, *sul2*, and *catB3*] predict resistance to beta-lactams, aminoglycosides, tetracycline, sulfonamides, and chloramphenicol (File S6). The presence of this GI on a plasmid underscores the dynamic role of MGEs in accelerating horizontal spread of resistance traits.

To identify shared and unique GIs across different strains, the genomes were analyzed using IslandCompare (Fig. 3D). The comparative analysis revealed both similarities and variations in GI clusters across all clades and subclades, highlighting the acquisition and loss of mobile GIs, as well as the genetic diversity and evolution of resistance. For example, the human-derived strains from China (QL588) and Taiwan (KT) shared 10 GI clusters, located in similar genomic regions, despite being isolated over a decade apart (QL588 in 2023 and KT in 2009). One of these shared GI clusters contained 32 genes associated with antibiotic resistance. Notably, the shared GI clusters were present across genomes regardless of the isolation source, year, or country, suggesting HGT and widespread dissemination of MGEs.

### Pathogenicity island prediction in *Morganella morganii* subspecies *morganii* strain KT

**Fig 4:**
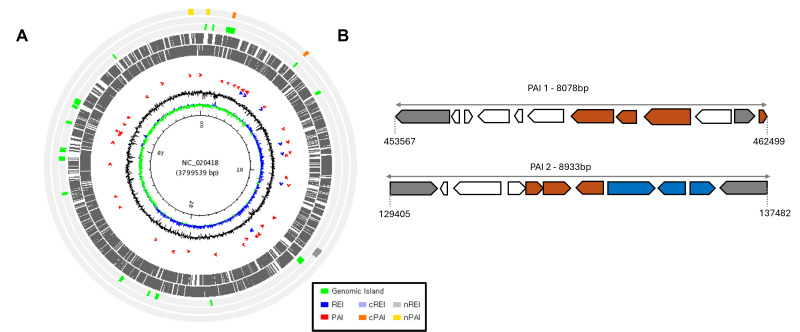
Pathogenicity and genomic island landscape of the *M. morganii* reference strain KT. (**A**) Circular map of the KT chromosome showing the distribution of predicted candidate pathogenicity islands (cPAIs, orange bars), non-probable PAIs (nPAIs, yellow bars), and genomic islands (GIs, green bars). Homologs of RGs (blue) and pathogen-associated genes (red) are indicated by colored circular glyphs inside the genome rings, while the inner circles represent G−C skew (blue-green) and G+C content (black). (**B**) Magnified view of representative genomic regions encoding cPAIs. Virulence genes (orange), RGs (blue), and MGEs (gray) are highlighted.

PAIs are a subset of GIs that encode virulence factors, facilitating bacterial infection and immune evasion. PAI prediction was performed only for the reference strain *M. morganii* KT (a human isolate from Taiwan) because the Pathogenicity Island Database (PAIDB) currently supports analysis exclusively for genomes already deposited and curated within its database, and KT is the only *M. morganii* genome available in PAIDB. PAIDB predicts PAIs, candidate PAIs, resistance islands (REIs), and candidate REIs across bacterial genomes. In KT, two candidate PAIs, two non-probable PAIs, and one resistance island outside of GIs were detected (Fig. 4A). These PAIs encode both resistance and virulence factors, some of which are already functionally characterized, while others are annotated as HPs (Fig. 4B). The predicted PAIs ranged from 8 to 15 kb and showed homology to known islands involved in virulence, resistance, and stress adaptation (40). According to PAIDB annotation, these include the Locus of Enterocyte Effacement, encoding a type III secretion system for host cell attachment (41); AGI-3, associated with acid resistance; the Tellurite Resistance-Associated Island; PAI V536, a uropathogenic *E. coli* island carrying adhesins and siderophores (42); PAI-I, involved in iron uptake; and the *Shigella flexneri* islands SHI-1, encoding enterotoxins, and SRL, a resistance locus for multiple antibiotics. These regions encode both resistance and virulence factors (Table 1).

**TABLE 1:** Profiles of pathogenicity islands in *Morganella morganii* subspecies *morganii* reference strain KT

Start	End	Size (kb)	ORFs	GC content (%)	No. of homologs (PAI-virulence genes)	PAIs homologous to this region	Insertion site	Virulence characteristics
129405	137482	8.78	12	40.39	4	SHI-1 (*S. flexneri 2a str. 301*)	tRNA *pheV*	Autotransporter family of proteins, enterotoxins of CT/LT-like toxin family, proteins exhibiting protease and cytopathic effect on host cells
						LEE (*E. coli* RW1374)	tRNA *pheV*	T3SS, intimin receptor, effector proteins
						AGI-3 (*E. coli BEN2908*)	tRNA *selC*	Putative mobile genetic elements, such as phage-related integrase genes and transposase genes
						TAI (*E. coli O157:H7 EDL933*)	Not known	Genes that mediate adherence to host cells, iron-regulated genes
						PAI V536 (*E. coli* 536)	tRNA *selC*	α-hemolysins, adhesin determinants, fimbriae
						PAI-I AL862 (*E. coli* AL862)	tRNA *pheR*	Adhesins and sugar utilization pathway proteins
453567	462499	8.93	11	40.11	3	*E. coli* EC20020119 SRL (*S. flexneri* YSH6000)	tRNA *serX*	Multiple antibiotic resistance determinants, siderophores, and prophages

### The resistome of *Morganella morganii*

**Fig 5:**
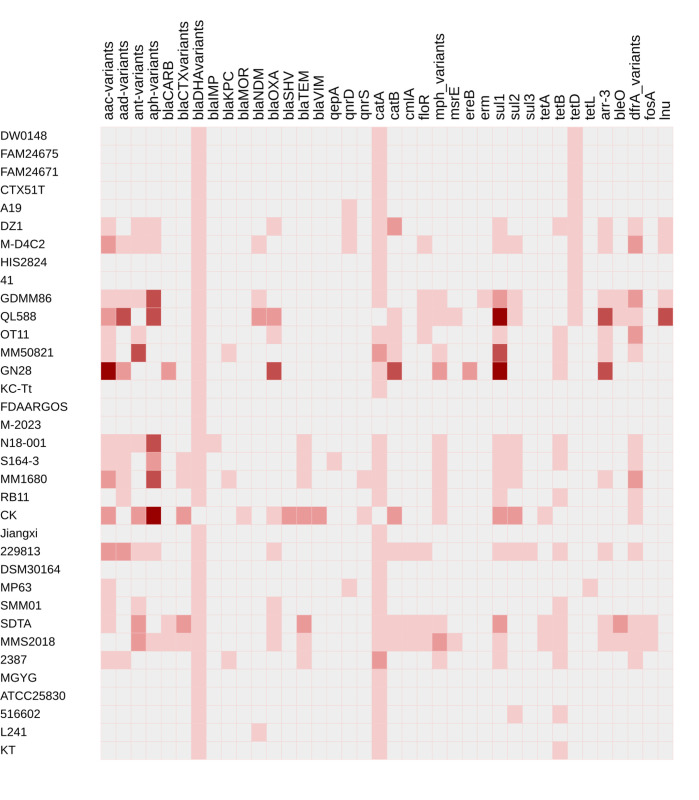
Resistome profile of 35 *M. morganii* strains. The heatmap displays the presence (shades of red) or absence (gray) of RGs. The different shades of red color indicate an increasing number of genes, from 1 (light red) to 6 (deep red), and the colorless squares represent the absence of the specific RGs in the strains. RGs are grouped by antibiotic class, including aminoglycosides, β-lactams, fluoroquinolones, chloramphenicol, macrolides, sulfonamides, tetracycline, rifampicin, bleomycin, trimethoprim, fosfomycin, and lincosamides. β-lactam RGs, particularly *bla_DHA_* variants and the chloramphenicol gene (*catA*), were the most frequently detected across strains. Strains QL588, GN28, and CK harbored the highest number of RGs. The absence of plasmids in GN28 is compensated for by other MGEs.

Analysis of predicted RGs revealed that the human-derived strain QL588 harbored the highest number of RGs (*n* = 33), followed by the wastewater-derived strain GN28 and another human-derived strain, CK, each with 30 RGs. Interestingly, while GN28 lacked plasmids, it appeared to compensate for this absence with other MGEs, whereas QL588 and CK possessed both plasmids and additional MGEs. In contrast, three human-derived strains (ATCC25830, MGYG, DSM30164), two animal-origin strains (Jiangxi and KC-Tt), and one food-derived strain (isolate 41) that lacked plasmids and transposable elements encoded only the *bla_DHA_* and *catA* genes (Fig. 5).

The first identified *M. morganii*, DSM30164, in 1905, only has resistance to β-lactams and chloramphenicol. The next identified *M. morganii* strain, KT, in 2009, has resistance to β-lactams, tetracycline, and chloramphenicol. Strains isolated in subsequent years appear to have acquired multiple RGs for different antibacterial classes, including currently used aminoglycosides and carbapenems. The *M. morganii* resistome predicted resistance to over ten antibiotic classes (Fig. 5). RGs against all classes of β-lactam antibiotics—particularly second- and third-generation cephalosporins, penicillins, monobactams, and carbapenems—were detected. The highest diversity of resistance genes was associated with resistance to β-lactam antibiotics, including *bla_DHA-13_, bla_DHA-21_, bla_DHA-17_, bla_DHA-11_, bla_DHA-16_, bla_DHA-4_, bla_DHA-18_, bla_CTX-2_, bla_MOR-2_, bla_VIM-1_, bla_NDM-1_, bla_OXA_*_-1_*, bla_KPC-2_,* and *bla_TEM_*. This was followed by chloramphenicol (*catA*), then aminoglycoside resistance genes, including *aac(3)-lld*, *aac(3)-lla*, *aac(3)-IV*, *aac(6*′*)-Ib-cr*, *aadA2*, *aadA1*, *aph(3*′*)-VI*, *aac(6*′*)-Ie-aph(2″)-Ia*, *aph(4)-Ia*, and *aadA16* (Fig. 5).

The most frequently detected RGs were variants of *bla_DHA_*, followed by a class C β-lactamase gene, chloramphenicol acetyltransferase genes (*catA*, *catB*), tetracycline efflux transporter RGs (*tetB*, *tetR*), and aminoglycoside RGs [*aph(3*′*)-Ia*, *aadA2*, *aac(3)-IV*]. Resistance genes conferring resistance to sulfamethoxazole (*sul1*, *sul2*), New Delhi metallo-β-lactamase (*bla_NDM-1_*), oxacillinase (*bla_OXA-1_*), and trimethoprim (dihydrofolate reductase; *dfr*) were also identified. Less frequently detected RGs included macrolide 2′-phosphotransferase [*mph(A*)] and erythromycin esterase (*ereB*), rifampicin resistance (rifampin ADP-ribosyltransferase; *arr-3*), fluoroquinolone resistance (*gyrA/gyrB* mutations, *qnrD*), lincosamide nucleotidyltransferase (*lnuF*), macrolide phosphotransferase (*msrE*), and fosfomycin resistance (*fosA8*; Fosfomycin thiol transferase).

Resistance mechanisms predicted by the Resistance Gene Identifier (RGI) tool in the Comprehensive Antibiotic Resistance Database (CARD) include antibiotic target alteration, antibiotic efflux, and antibiotic inactivation. Detected efflux pump genes included resistance-nodulation-cell division family pumps (*crp*, *rsmA*), small multidrug resistance (SMR) proteins (*qacG*), and major facilitator superfamily (MFS) efflux pumps (*smr*, *mfs*).

### TA systems in *Morganella morganii*

**Fig 6:**
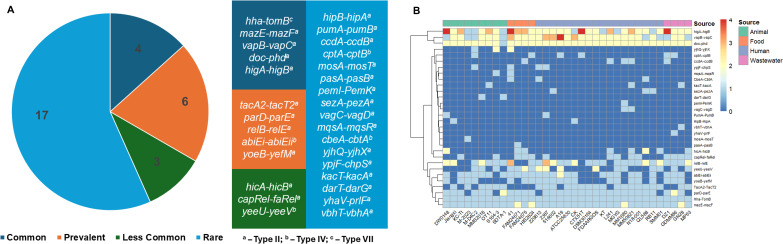
Diversity and distribution of toxin-antitoxin systems in *M. morganii*. (**A**) TA systems were classified based on their frequency across strains as common (≥90%), prevalent (50%–90%), less common (20%–50%), or rare (<20%). (**B**) Heatmap showing the presence and absence of TA systems across 35 *M. morganii* strains isolated from diverse sources. Each row represents a unique TA system pair, while each column represents a bacterial strain. TA system presence is color-coded from 0 (absent; dark blue) to 4 (high copy number or multiple loci; red), based on detection counts. Strains are grouped by source animal, food, human, and wastewater, indicated by the colored annotation bar at the top. Hierarchical clustering was applied to both strains and TA systems based on binary presence/absence profiles to highlight patterns of distribution. Note that orphan TA genes are not included in the analysis for this figure.

TA systems were predicted using TADB 3.0, identifying both paired and orphan (unpaired toxins or unpaired antitoxins). TAs belonging to the type II, IV, and VII classes. Most strains carried abundant TAs, with a minimum of 10 paired and 83 unpaired TAs per genome, independent of source or presence of MGEs. Of the 30 predicted TA gene families, 25 were type II, 4 were type IV, and 1 was type VII. Notably, the more recently discovered DarTG and ToxSAS (CapRel, FaRel) systems were also identified (30, 43). TA gene families were categorized based on their frequency across strains: common (≥90%), prevalent (50%–90%), less common (20%–50%), and rare (<20%). Based on these criteria, only five TA families were common, and five were prevalent to all strains, while three TA families were less common and 17 others appeared in less than 20 percent of the strains (Fig. 6A). The TAs that appear multiple times in at least one of the strains include *higB-higA, mazE-mazF, parD-parE, relB-relE, yeeU-yeeV, hicB-hicA, AbiE, VapBC, yjhQ-yjhX,* and *doc-phd* (Fig. 6B, blue to red color scale). Strain M-2023 (animal origin) had the fewest paired TAs (10), while strain 41 (food origin) had the most (23), followed by strain 2387 (human origin, 21 TAs). About 11 strains harbored over 100 orphan TAs, with strain DW0548 (animal origin) having the highest (111). Strains lacking plasmids and transposable elements still encoded 11–23 paired and 79–89 orphan TAs, highlighting the chromosomal abundance of TAs (File S7). However, the presence of MGEs correlates with an increased number of TAs, especially orphan TA genes.

Functionally, the common TA pairs found in nearly all strains (*hha-tomB, higA-higB, mazE-mazF, vapB-vapC,* and *doc-phd*) are primarily associated with stress survival, translation inhibition, and regulation of programmed cell death (44, 45). Prevalent TAs included *parD-parE, relB-relE, yoeB-yefM, tacA2-T2,* and *abiE-abiEi*, known for their roles in DNA replication inhibition, mRNA cleavage, and phage resistance (46, 47). Less common TAs (*hicA-hicB, capRel-faRel,* and *yeeU-yeeV*) are involved in translation inhibition and persistence under stress (48, 49). Several TA systems were classified as rare, including *hipB-hipA, pumA-pumB, ccdA-ccdB, cptA-cptB, mosA-mosT, pasA-pasB, pemI-pemK, sezA-pezA, vagC-vagD, mqsA-mqsR, CbeA-CbtA, yjhQ-yjhX, ypjF-chpS, kacT-kacA, darT-darG, yhaV-prlF*, and *vbhT-vbhA*, many of which are associated with growth inhibition, phage defense, stress adaptation, translation or replication interference, and antibiotic persistence (50–52) (Fig. 6A).

## DISCUSSION

Recent studies have shown that *M. morganii* is increasingly becoming a public health threat, with cases of infections on the rise (3). Most of the infections caused by *M. morganii* are difficult to treat due to resistance to third-generation cephalosporins and carbapenems (53). There is therefore a need to understand the genomic features that may contribute to the pathogenicity, adaptability, and AMR of *M. morganii* to inform treatment strategies. This study profiled 35 *M. morganii* strains deposited in NCBI isolated from humans, animals, food, and wastewater with a goal of characterizing the diversity of virulence-associated factors, specifically GIs, RGs, and TAs, that could potentially contribute to *M. morganii* pathogenicity. Although the data set includes isolates spanning multiple continents, the predominance of genomes from China highlights a geographic sampling bias consistent with current trends in public databases. This imbalance emphasizes the need for expanded genomic surveillance across underrepresented regions to more comprehensively capture the global genomic diversity and evolutionary dynamics of *M. morganii*.

Using *in silico* data analysis, diverse virulence factors within the genomes of *M. morganii* were identified. Most of these factors were associated with MGEs and were either inside an MGE, overlapped with an MGE, or flanked by one or two MGEs. This genomic organization suggests that HGT plays a central role in shaping the accessory genome of *M. morganii*, facilitating the acquisition and dissemination of resistance and virulence determinants. These MGEs can be generated from plasmids, transposons, integrons, GIs, and phages, and are known to be involved in the evolution and propagation of RGs (54). Their widespread distribution across *M. morganii* genomes irrespective of the isolation source, year, or geographic region validates the view that genome plasticity is a conserved and ongoing feature of this species. Consistent with previous reports implicating integrative conjugative elements (ICEs) and plasmids in the acquisition of resistance and virulence traits in *M. morganii* (38–40)*,* our findings position MGEs as a primary evolutionary mechanism underlying the adaptive potential of this organism.

Comparative analysis across isolates from clinical, animal, food, and environmental sources revealed both shared and source-specific genomic features. Plasmids and other MGEs were unevenly distributed: clinical and animal isolates generally carried more MGEs, whereas food and wastewater strains were more variable. All isolates carried at least one key RG, though strains with plasmids or MGEs harbored a higher number of RGs, highlighting the role of mobile elements in multi-resistance. TA systems were present in all strains, independent of plasmid or MGE content, suggesting that they are core genomic features. Genomic islands were detected in all genomes, with their number positively associated with plasmid and MGE abundance, consistent with horizontal gene transfer shaping genome architecture. These observations should be interpreted with caution, given the overrepresentation of clinical isolates in this study set. Nevertheless, they collectively support a model in which genome plasticity and MGE-mediated gene flow contribute substantially to the pathogenic and adaptive landscape of *M. morganii*.

A considerable proportion of the *M. morganii* core genome (approximately 24%) consists of genes annotated as HPs, highlighting a significant gap in functional characterization even among conserved genomic elements. These HPs span a broad size range, with over one-third falling between 100 and 200 amino acids and approximately 12% classified as small proteins (≤100 amino acids). Small proteins are known to contribute to various biological processes, including signaling, transport, metabolic pathways, regulation, and response to environmental stresses (55) and are increasingly explored as therapeutic targets (56). Rather than representing functionally uncharacterized regions, re-annotation suggests that many of these proteins correspond to divergent members of established protein families, indicating functional conservation despite sequence divergence. The association of several hypothetical proteins with pathways linked to secretion systems, two-component regulation, AMR, and TA modules suggests that these proteins may contribute to adaptive and pathogenic processes rather than being limited to basic cellular maintenance.

The persistence of a subset of HPs despite extensive homology- and domain-based annotation raises the possibility of lineage-specific functions that may be unique to *M. morganii* or closely related taxa. Their conservation within the core genome, together with their frequent localization near MGE and pathogenicity-associated regions, points to potential roles in genome plasticity, stress tolerance, or host interaction. Similar observations where conserved HPs contribute to pathogenicity and stress adaptation have been reported in *Corynebacterium pseudotuberculosis* (57) and *Bacillus paralicheniformis* (58), supporting the biological relevance of these findings. Overall, these HPs represent a reservoir of underexplored functions that may shape the adaptive capacity and pathogenic potential of *M. morganii* and warrant future experimental investigation.

Genomic island analysis revealed clusters ranging from approximately 10 to 167 kb frequently integrated near tRNA loci and enriched in MGEs, virulence factors, and RGs indicative of pathogenicity islands. The integration near tRNA genes is indicative of HGT-acquired GIs, and the presence of MGEs suggests the transfer could be through plasmids, transposons, or bacteriophages. This highlights not only the mobility and stability of these islands but also their role in promoting genetic diversity and the evolution of AMR. Nearly all strains have clusters encoding RG, stress-response proteins, or PAPs, often in combination with TA systems. The recurrent co-localization of TA modules within these islands suggests a stabilizing role, consistent with previous reports demonstrating TA-mediated maintenance of GIs (31, 59). In addition to this structural role, several type II TA systems, including *darT-darG*, *abiEi-abiEii*, and *pemI-pemK,* have been shown to mediate anti-phage defense in *E. coli, M. tuberculosis*, *Serratia* sp., and *K. pneumoniae* (30, 60). The presence of anti-phage TA systems, such as DarTG and AbiE, within *M. morganii* genomic islands suggests a potential role in limiting phage infection. Notably, prophages and phage-associated genes are also detected in these genomes, which does not contradict this interpretation. Anti-phage TA systems are often phage-specific or conditionally active and do not necessarily confer complete resistance. Their coexistence with phage elements likely reflects sustained phage-mediated selective pressure, highlighting ongoing interactions between bacteria and their viral predators. The identification of characterized *M. morganii* phages, such as FSP1, which influences bacterial growth and host-associated traits (7, 34, 36), further supports the ecological relevance of bacteriophages in shaping the genomic composition of this species.

The largest GI cluster identified (~167 kb) in the environmental strain GDMM86 harbors multiple copies of *sul1, qacE, Arr-3, catB, bla_NDM-1_, ble, mphA, aph(3*′*),* and *mrxA,* suggesting resistance to about seven classes of antimicrobials, such as carbapenems. The density and diversity of resistance determinants within this single island exceed those reported in well-characterized MDR GIs, such as *Salmonella* GI 1 (61), highlighting the potential of *M. morganii* to act as a reservoir for complex resistance assemblies. In this study, the RG cargo on the GI cluster was preceded by either an insertion-like element transposase, Tn3 family transposase, class 1 integron integrase, or a recombinase, and other ICE proteins. The association of resistance loci with integrases, transposases, recombinases, and ICE components suggests that this island is highly mobile and may have been acquired through interspecies gene transfer, analogous to resistance- and virulence-associated PAIs in other Enterobacterales, including *Enterococci* (62), *Serratia* sp. (63), *S. enteritica* (64), and *M. morganii* (65). In addition to resistance genes, this GI cluster encoded prophages, stress-response genes, GNAT family acetyltransferases, and multiple type II TA systems, including Prlf-YhaV and DUF-containing putative TA pairs. Type II TA systems have been implicated in antibiotic and stress tolerance of pathogenic bacteria, including *E. coli* and *Salmonella,* via a persistence-inducing mechanism (66–68). Our study is the first to identify and catalog TAs in different *M. morganii* strains. Together with prior reports of TA-containing GIs in uropathogenic and environmental *M. morganii* isolates (15, 69), our findings support a model in which GIs function as integrated adaptive platforms that couple AMR, stress tolerance, and genome stabilization. Although experimental validation is required, these genomic features generate testable hypotheses regarding the roles of TA systems and HPs in shaping the pathogenic and ecological success of *M. morganii*.

The presence and absence of plasmids and other MGEs play a key role in the acquisition and maintenance of the RGs over time in *M. morganii*. Strains lacking plasmids and transposable elements carried only chromosomally encoded resistant determinants, such as *bla_DHA_* and *catA* genes, whereas strains harboring MGEs encoded resistance to ten or more antimicrobial classes. These genomic profiles are consistent with recent reports of MDR *M. morganii* phenotypes (3, 20, 70), reinforcing the link between genome plasticity and AMR. In the current study, an array of acquired RGs encoded on chromosomes and plasmids was identified in selected *M. morganii* strains. The β-lactam resistance class had the highest diversity of RGs (*n* = 14), followed by aminoglycosides (*n* = 10). Notably, clinically important carbapenemase genes, including NDM-1, KPC-2, OXA-48, and DHA, have been recently reported in *M. morganii* (69, 71, 72), similar to findings in our study.

In parallel, *M. morganii* genomes harbored an abundance of TAs borne on plasmids, insertion-like elements, and chromosomes. There were 30 distinct TA families, predominantly belonging to the type II group, except for four TA systems that were classified as type IV and one as type VII. The ubiquitous presence of the *hha-tomB* system, despite the absence of functional characterization in *M. morganii,* is notable given its established role in persister formation and virulence regulation in *E. coli* and *S. typhimurium* (73–75). Similarly, the frequent occurrence of *higBA* and *vapBC* systems, often in multiple copies and within mobile contexts, suggests selective retention of these modules, potentially due to their roles in stress tolerance and persistence, as reported in other bacteria (28, 32). The identification of *higBA* on plasmids carrying resistance and virulence-associated genes further supports a functional link between TA systems and adaptive traits.

The current co-localization of RGs, TA systems, and virulence-associated genes within transposons, integrons, prophages, and GIs indicates a coordinated architecture that facilitates HGT while promoting genomic stability. TA systems are known to enhance plasmid and GI maintenance (59, 76, 77), and their consistent association with MGEs in *M. morganii* suggests a similar stabilizing role, enabling the long-term persistence of resistance and virulence determinants. The presence of prophage-associated resistance and virulence genes further implies that phage-mediated transduction may contribute to gene flow within *M. morganii* populations. Comparable observations have been reported in other bacterial species: for example*, higBA* in *Burkholderia pseudomallei* is located within a prophage alongside a transposase (28), *vagCD* in *Klebsiella pneumoniae* resides on a conserved plasmid encoding resistance genes, transposases, relaxases, PAPs, and stress-response genes (78), and *CapRel*, a fused TA in the GI of *Mycobacterium terramassiliense*, inhibits protein synthesis and induces growth arrest (79).

Collectively, our findings support a model in which *M. morganii* functions as a highly plastic opportunistic pathogen whose adaptability is driven by extensive HGT. Mobile genetic elements act as dynamic vehicles for the acquisition and dissemination of AMR and virulence determinants, while TA systems and conserved HPs likely contribute to genome stabilization, stress tolerance, and persistence under selective pressures. This integrative genomic framework provides testable hypotheses regarding the interplay between MGEs, TA systems, and resistance evolution, and establishes a foundation for future experimental studies examining bacterial survival under environmental and antimicrobial stress. The conserved and recurrent nature of these elements across diverse isolates underscores their potential importance in shaping the ecological success and pathogenic potential of *M. morganii*.

## MATERIALS AND METHODS

### Genome data collection and comparative analysis

Complete genome sequences of 35 *M. morganii* strains (Table S1) were retrieved from the NCBI RefSeq database and used for comprehensive genomic analyses. All genomes were analyzed using standardized pipelines for the identification of plasmids, MGEs, RGs, TA systems, and GIs, ensuring uniformity of analysis despite differences in original sequencing and assembly methods.

### Pangenome and phylogeny construction

To characterize the pan-genome, Panaroo v1.5.0 (38), which clusters genes from all genomes in the data set, was used. The --strict flag was applied to ensure that only high-confidence gene sequences were included in the analysis. The core genome alignment, derived from Panaroo, was then used to infer the phylogenetic relationships among the strains. A maximum likelihood phylogenetic tree was constructed using IQ-TREE multicore v2.3.6 (80). The alignment file (core_gene_alignment.aln) was used as input, and the best-fit substitution model: generalized time-reversible (GTR+G) was selected for the analysis. Bootstrap support was calculated using 1,000 bootstrap replicates (-bb 1000), and 1,000 ultrafast bootstrap replicates were performed for branch support (-alrt 1000). The resulting phylogenetic tree was visualized using the Interactive Tree of Life (iTOL) (81) for further analysis and presentation.

### Identification of MGEs

Plasmids and transposable elements present in the selected *M. morganii* strains were predicted by MobileElementFinder v1.0.2 (82). The plasmid typing was further confirmed using PlasmidFinder in Abricate v1.0.1 (83). Sequences of the MGEs were extracted, concatenated, and annotated using Bakta v1.1.0 via Proksee v1.1.3 (84). Bakta uses multiple external tools, including Prokka, EggNOG, Pfam, MetaPhlAn, and the Bacterial Antimicrobial Resistance Database. It integrates annotations from multiple sources to improve functional predictions. GIs were predicted using Alien-Hunter v1.7 (85) and compared on IslandCompare v1.0 (86) using default parameters. IslandCompare performs a phylogenetic analysis and alignment of the genomes and predicts GIs using integrated GI prediction software—IslandPath-DIMOB and Sigi-HMM. The pathogenicity islands database (http://www.paidb.re.kr/about_paidb.php?m=h) was used for further characterization of the KT strain (40). PAI predictions were not uniformly applied across all analyzed strains because PAIDB supports analysis exclusively for genomes already deposited and curated within its database, and KT is the only *M. morganii* genome available in PAIDB. Predicted GI regions were extracted based on coordinate files and annotated with Bakta and Prokka v1.14.6. Functional categorization of genes within GIs was performed by screening for virulence factors (VFDB) in Abricate, RGs (CARD), MGEs (mobile OG-db), and TA systems (TADB3.0). Visualization was done using Proksee v1.1.3.

### Genomic profiling of antimicrobial RGs in *Morganella morganii*

To identify resistance genes and antimicrobial classes, the genomes of the selected *M. morganii* strains (File S1) from the NCBI database were submitted to ResFinder (https://cge.food.dtu.dk/services/ResFinder/) and the resistance gene identifier (https://card.mcmaster.ca/analyze/rgi) on CARD. The ResFinder (v4.5.0) database predicts phenotype from genotype using 15 antimicrobial drug classes and 2,690 RGs (87). The genes available in the database were BLASTed against the whole-genome sequence of the bacteria using default criteria for chromosomal point mutations and acquired RGs (90% threshold for percentage identity and 60% minimum length). The percentage identity was the number of identical nucleotides that best match the resistance gene and the equivalent sequence in the *M. morganii* genome. The minimum length of the matching gene is the number of nucleotides that cover a resistant gene. The RG identifier (RGI) online software predicts resistance determinants based on homology and SNP models (88). The best matching hits were generated as either perfect, strict, or loose; and in this study, only perfect (100% match to a reference) and strict (RGs with mutations) hits were considered.

### Identification of TA systems in *Morganella morganii*

The different TA systems in *M. morganii* were identified by submitting the nucleotide sequences of each genome (chromosomes and MGEs) to TADB3.0 (https://bioinfo-mml.sjtu.edu.cn/TADB3/). TADB3.0 leverages the TAfinder 2.0 prediction tool and identifies TA loci that belong to all TA types; I to VIII (89). TADB3.0 searches only genes with feature characteristics of TA systems to minimize false positives (90).

## Data Availability

All data generated are included in the article. Complete genome sequences of *Morganella morganii* strains analyzed in this study were retrieved from NCBI and are available as Supplemental material.

## SUPPLEMENTAL MATERIAL

Supplemental material spectrum.02207-25-s0001.xlsx Files S1 to S6.

ASM does not own the copyrights to Supplemental Material that may be linked to, or accessed through, an article. The authors have granted ASM a non-exclusive, world-wide license to publish the Supplemental Material files. Please contact the corresponding author directly for reuse.
